# Multiple locus VNTR analysis highlights that geographical clustering and distribution of *Dichelobacter nodosus*, the causal agent of footrot in sheep, correlates with inter-country movements^☆^

**DOI:** 10.1016/j.meegid.2013.05.026

**Published:** 2014-03

**Authors:** Claire L. Russell, Edward M. Smith, Leonides A. Calvo-Bado, Laura E. Green, Elizabeth M.H. Wellington, Graham F. Medley, Lynda J. Moore, Rosemary Grogono-Thomas

**Affiliations:** a Department of Clinical Veterinary Sciences, University of Bristol, Langford House, Langford, Bristol BS40 5DU, England, United Kingdom; b School of Life Sciences, University of Warwick, Gibbet Hill Road, Coventry CV4 7AL, England, United Kingdom

**Keywords:** *Dichelobacter nodosus*, Global distribution, MLVA, Ovine footrot

## Abstract

*Dichelobacter nodosus* is a Gram-negative, anaerobic bacterium and the causal agent of footrot in sheep. Multiple locus variable number tandem repeat (VNTR) analysis (MLVA) is a portable technique that involves the identification and enumeration of polymorphic tandem repeats across the genome. The aims of this study were to develop an MLVA scheme for *D. nodosus* suitable for use as a molecular typing tool, and to apply it to a global collection of isolates. Seventy-seven isolates selected from regions with a long history of footrot (GB, Australia) and regions where footrot has recently been reported (India, Scandinavia), were characterised. From an initial 61 potential VNTR regions, four loci were identified as usable and in combination had the attributes required of a typing method for use in bacterial epidemiology: high discriminatory power (*D* > 0.95), typeability and reproducibility. Results from the analysis indicate that *D. nodosus* appears to have evolved via recombinational exchanges and clonal diversification. This has resulted in some clonal complexes that contain isolates from multiple countries and continents; and others that contain isolates from a single geographic location (country or region). The distribution of alleles between countries matches historical accounts of sheep movements, suggesting that the MLVA technique is sufficiently specific and sensitive for an epidemiological investigation of the global distribution of **D. nodosus**.

## Highlights

• Development of a portable MLVA technique to characterise *Dichelobacter nodosus*.
• Seventy-seven isolates from three continents typed.
• Isolates from countries with a long and short history of footrot.
• Allele distribution between countries matches historical accounts of sheep movement.
• *D. nodosus* has evolved via recombinational exchanges and clonal diversification.

## 1 Introduction

Footrot and interdigital dermatitis are the most common causes of lameness in sheep in the UK (Grogono-Thomas and Johnston, 1997; Kaler and Green, 2009; Wassink et al., 2003, 2004); and footrot has been estimated to cost the sheep industry in Great Britain alone approximately £24.4  million per year (Nieuwhof and Bishop, 2005). The principal causal agent of both presentations of disease is the Gram-negative, anaerobic bacterium *Dichelobacter nodosus*. The pathogenesis of footrot begins with a reduction in the structural integrity of the interdigital skin. The epidermis is then invaded by *D. nodosus* which perturbs the microbial community (Calvo-Bado et al., 2011) and disease develops as the load of *D. nodosus* increases (Witcomb, 2012). *D. nodosus* digests the epidermis of the foot, a process associated with the secretion of several serine proteases (Kennan et al., 2010) resulting in necrosis and inflammation. This can lead to separation of the hoof horn from the underlying epidermis, causing lameness, which if left untreated can persist for many weeks, causing chronic lameness (Beveridge, 1941).

Footrot, has been described on every continent except Antarctica (Aguiar et al., 2011; Azizi et al., 2011; Buller et al., 2010; Dold and Cocks, 2001; Eze, 2002; Ghimire et al., 1998; John et al., 1999; Morck et al., 1994; Wani et al., 2004; Zakaria et al., 1998). Early accounts of the disease date back to the 15th century in Europe ((Youatt, 1837) http://wakeringheritage.org.uk/historybycentury.html, last accessed 26th March 2013) and the 19th century in Australia (Graham, 1870). Anecdotal evidence suggests that the disease was introduced into India following the importation of Australian Corriedale, Merino and Rambouillet sheep as part of a genetic improvement program in the 1970s (Dixit et al., 2006; Mittal and Ghosh, 1979). More recently footrot has been detected in Norway and Sweden (Meling and Ulvund, 2009; Olofsson et al., 2005; Øverås, 1994), countries where it had previously been considered absent/eliminated.

To date, molecular analyses of *D. nodosus* have focused on analysis of isolates from a single country and included pulsed-field gel electrophoresis (PFGE), infrequent restriction site PCR (IRS-PCR) and restriction fragment length polymorphism (RFLP) approaches (Buller et al., 2010; Ghimire and Egerton, 1999; Zakaria et al., 1998). These methods can be slow, costly, labour-intensive and can lack reproducibility between laboratories and over time. Multiple-locus variable number tandem repeat (VNTR) analysis (MLVA) characterises the diversity of polymorphic tandem repeats located throughout the genome. The variation in number of tandem repeats at a given locus is associated with slipped strand mispairing during DNA replication (Levinson and Gutman, 1987), and characterising this variation permits the discrimination of bacterial isolates. Comparison of MLVA profiles can provide information on bacterial evolution and population diversity and may allow for hypothesising routes of geographical dissemination. MLVA has proved successful in epidemiological typing of pathogenic bacteria including *Streptococcus agalactiae*, *Staphylococcus aureus*, *Listeria monocytogenes* and *Escherichia coli* (Chen et al., 2011; Haguenoer et al., 2011; Noller et al., 2003; Sobral et al., 2012).

The aims of the current study were to identify polymorphic loci in the *D. nodosus* genome, test these to develop an MLVA scheme suitable for use as an epidemiological tool and to type a global collection of *D. nodosus* isolates to investigate the global distribution of strains of *D. nodosus*.

## 2 Materials and methods

### 2.1 *D. nodosus* isolates

Seventy-seven *D. nodosus* isolates were analysed from Australia (*n* = 29), GB (*n* = 22), India (*n* = 15), Norway (*n* = 7) and Sweden (*n* = 4; Table S1). In addition to thermostable and thermolabile control isolates and those representing known serogroups, isolates were selected based on broad geographical distribution from regions with a long history of footrot (GB, Australia) and those where footrot has recently been reported (Scandinavia, India). Isolates were cultured on 4% hoof agar (1% [w/v] proteose peptone [No. 3, Difco], 0.5% [w/v] NaCl, 0.4% [w/v] beef extract [Oxoid, Lab-Lemco], 0.1% [w/v] yeast extract [Sigma], 1.5% [w/v] finely ground ovine hoof horn (ovine hoof material was removed from sheep feet post-mortem and ground to a fine powder by SAC Analytical Services Dept., Edinburgh), 4% [w/v] Bacto agar [Difco]) (Thomas, 1958; Thorley, 1976) at 37 °C for three days under anaerobic conditions (AnaeroGen, Oxoid Ltd., UK). Genomic DNA was extracted using the Nucleospin® Blood Kit (Macherey–Nagel, Düren, Germany) following the manufacturer’s instructions for DNA extraction from Gram-negative organisms.

### 2.2 Identification of VNTRs

At the time of development, the complete genome sequence was only available for *D. nodosus* VCS1703A (Myers et al., 2007). Therefore we were unable to compare tandem repeat loci between complete genomes of multiple isolates. The genome sequence of *D. nodosus* VCS1703A (GenBank Accession number CP000513) was analysed using the Tandem Repeats Finder software v.4.04 (Benson, 1999) [http://tandem.bu.edu/trf/trf.html, last accessed 26th March 2013]. The selection criteria for potential MLVA markers were a consensus sequence sequence ⩾ 5 bp and copy number number ⩾ 2 in the VCS1703A strain.

### 2.3 PCR amplification

PCR primers targeting the 5’and 3’ flanking regions of each tandem repeat locus were designed using Primer3 software (v.0.4.0; http://frodo.wi.mit.edu/primer3/, last accessed 26th March 2013) and produced by Metabion International AG (Martinsried, Germany). PCR cycling conditions for all tandem repeat loci were 95 °C for 2 min, 30 cycles of 94 °C for 1 min, 59 °C for 30 s and 72 °C for 1 min and a final extension of 72 °C for 2 min. Each reaction contained 25 μl 2 × PCR master mix (Promega), 1.0 μl forward and reverse primer [10 mM], 1.0 μl DNA template, 2.5 μl DMSO and 2.0 μl BSA [100 mg/ml] in a final volume of 50 μl. Proline-glycine repeat (Pgr) status was determined as described previously (Calvo-Bado et al., 2011). A multiplex PCR was used to determine *D. nodosus* serogroup based on Dhungyel et al. (2002), with modifications for use with genomic DNA. Briefly, a common forward primer was used with three groups of reverse primers (ABC; DEF; GHI). PCR cycles were 95 °C for 15 min, 5 cycles of 94 °C for 30 s, 60 °C for 30 s, 72 °C for 30 s, followed by 25 cycles of 94 °C for 30 s, 58 °C for 30 s, 72 °C for 30 s. Each reaction contained 12.5 μl Go-Taq Hotstart (Promega), 4.75 μl BSA [0.28 mg/ml], 2.5 μl MgCl_2_ [50 mM], 3.0 μl forward primer [10 μM], 1.0 μl each reverse primer (ABC; DEF; GHI; 10 μM each) and 1.0 μl DNA template in a final volume of 25 μl. All amplifications were performed on an MJ Research200 thermocycler (Bio-Rad, Hertfordshire, UK), and PCR amplicons visualised on 1% and 2% agarose gels containing ethidium bromide under UV light using a Bio-Rad Gel Doc 2000 imager (Bio-Rad Laboratories Ltd., Hertfordshire, UK).

### 2.4 Sequencing

VNTR amplicons were purified using the NucleoSpin® Extract II Kit (Macherey–Nagel, Düren, Germany) as recommended by the manufacturer, and submitted for sequencing with the forward primer to The Sequencing Service (School of Life Sciences, University of Dundee, UK). All sequences were determined using Applied Biosystems BigDye v.3.1 chemistry on an Applied Biosystems 3730 automated capillary DNA sequencer (Applied Biosystems, Foster City, CA, USA). Sequence data were analysed using MacVector® with assembler, v.9.5.2 (MacVector Inc, Cary, NC, USA). Individual ABIview sequence files were analysed using the Phred function and any poor quality data files re-sequenced.

### 2.5 Gelatin gel for protease thermostability

The gelatin gel assay was used to determine protease activity as described previously (Moore et al., 2005; Palmer, 1993).

### 2.6 MLVA stability

The stability of the *D. nodosus* MLVA scheme was tested by passaging three *D. nodosus* isolates (VCS1703A, C305 and 18e) on Wilkins-Chalgren Anaerobe agar (CM0619; Oxoid, Basingstoke, UK) and 4% hoof agar for a total of 30 passages. MLVA profiles were determined every 10 passages.

### 2.7 Data analysis

The MLVA allelic profile was determined for each isolate based on the number of repeats at each of the four chosen loci: DNTR02, DNTR09, DNTR10 and DNTR19. Unique MLVA allelic profiles were arbitrarily assigned a single numerical genotype identifier (designated an MLVA type), and this dataset was used in the analyses described below.

### 2.8 Diversity index and linkage disequilibrium

Simpson’s index of diversity [*D*] (Simpson, 1949) were calculated for individual and combined loci using V-DICE (VNTR DIversity and Confidence Extractor; http://www.hpa-bioinformatics.org.uk/cgi-bin/DICI/DICI.pl, last accessed 26th March 2013).

### 2.9 Population analyses

The global optimal eBURST (goeBURST) algorithm (Francisco et al., 2009), implemented in PHYLOViZ (Francisco et al., 2012) was used to divide the *D. nodosus* population into clonal complexes (CCs) containing single locus variant (SLV) relationships. CCs were named on the basis of the predicted ancestral strain(s). These were combined into a minimum-spanning tree (MST) by inclusion of double locus variant (DLV) relationships. Population structure was also analysed using Structure v2.3.4 (Falush et al., 2003; Pritchard et al., 2000), a Bayesian model-based clustering approach that divides the population into *K* user-defined independent clusters. The range of *K* values tested was 1–11. For each value of *K* we performed 10 runs with a burn-in period of 10^5^ and then 10^5^ MCMC steps. All runs used the admixture model and assumed allele frequencies were correlated among populations. The true number of clusters (*K*^∗^) at the uppermost hierarchical level of population structure was determined by calculating *ΔK* using the ‘Evanno method’ (Evanno et al., 2005) implemented in Structure Harvester (Earl and vonHoldt, 2012). Graphical outputs of Structure runs were visualised using Clumpp (Jakobsson and Rosenberg, 2007) and Distruct (Rosenberg, 2004). The presence of linkage disequilibrium (LD) within the population was tested using Monte-Carlo and parametric tests with 1,000 random resamplings, implemented in Lian v.3.5 (Haubold and Hudson, 2000). The NeighbourNet function of SplitsTree4 v.4.11.3 (Huson and Bryant, 2006) with default settings was used to construct a phylogenetic network to describe the population evolutionary history of the analysed strains.

## 3 Results

### 3.1 Descriptive results

Of the 77 isolates, 50 were thermostable (virulent) and 27 thermolabile (benign) (Table 1). All Indian and Norwegian isolates were thermostable whereas all four Swedish isolates were thermolabile. The populations from Australia and GB contained both thermostable and thermolabile isolates (Table 1). The *pgr* gene was detected in all isolates, *pgrA* was present in 45 isolates (58%), and *pgrB* in 32 (42%). Of the 45 *pgrA* isolates, 44 (97.7%) were thermostable and 26/32 (81.3%) *pgrB* isolates were thermolabile (Table 1). In ovine isolates from Australia, India, Norway and Sweden, all *pgrA* variants were thermostable, and all *pgrB* variants were thermolabile; however isolates from GB were more variable: some *pgrB* variants were classed as thermostable (Supplementary dataset 1). The Swedish *pgrA* isolate (thermolabile) was originally isolated from a cow. PgrA was present in ovine isolates from Australia, GB, India and Norway, and PgrB from Australia, GB and Sweden. There were nine serogroups among the 77 isolates; serogroup A was the most prevalent (21/77; 27.3%), and serogroup D the least prevalent (1/77; 1.3% [control isolate]). There was no apparent relationship between serogroup and MLVA profile or individual alleles at each locus.

### 3.2 Selection of variable number tandem repeat loci

Sixty-one tandem repeat regions were identified in the *D. nodosus* VCS1703A genome. Of these, 34 fitted the selection criteria (consensus sequence sequence ⩾ 5 bp, and copy number number ⩾ 2) and were identified as potential MLVA markers. The 34 loci were initially characterised in eight geographically diverse isolates (Supplementary dataset 1) to determine the degree of polymorphism. From these initial analyses, four loci were selected for use in the MLVA scheme (Table S1) based on ease of amplification and not located in *vap* or *vrl* (Haring et al., 1995; Katz et al., 1992) virulence associated regions because these genomic loci are not present in all isolates of *D. nodosus* (Rood et al., 1996). Additional reasons for discarding loci included lack of polymorphism, inability to amplify the region or changes in tandem repeat consensus sequence between isolates (Table S2).

### 3.3 Stability of the *D. nodosus* MLVA loci

There was no variation in the profiles of the passaged isolates.

### 3.4 Population diversity

The four MLVA loci (designated DNTR02, DNTR09, DNTR10 and DNTR19) were characterised in all 77 *D. nodosus* isolates and the number of repeats present in each strain determined to the nearest full consensus sequence. The overall level of diversity detected (Simpson’s *D*) was 0.969, and ranged from 0.504 to 0.937 for individual loci (Table 2). DNTR09 and DNTR19 have the same number of alleles (*n* = 4), however two DNTR09 alleles are present in 96.1% (*n* = 74) of the isolates investigated, whereas all alleles of DNTR19 are present in at least seven (9.0%) isolates. The difference in allelic distribution means that DNTR19 is considered marginally more diverse. Examination of the allelic distributions of DNTR09 and DNTR19 at the country level (Table 3) reveals some interesting patterns. Within DNTR09, allele05 is present in every country studied, however allele04 was not present in the isolates from India or Norway. At locus DNTR19, no allele is present in every country studied; allele02 is unique to Indian isolates, allele03 is not present in the Scandinavian isolates, allele04 was only detected in Australia and GB, and allele05 was present in Australia, GB, Norway and Sweden.

### 3.5 Population structure

**Fig. 1:**
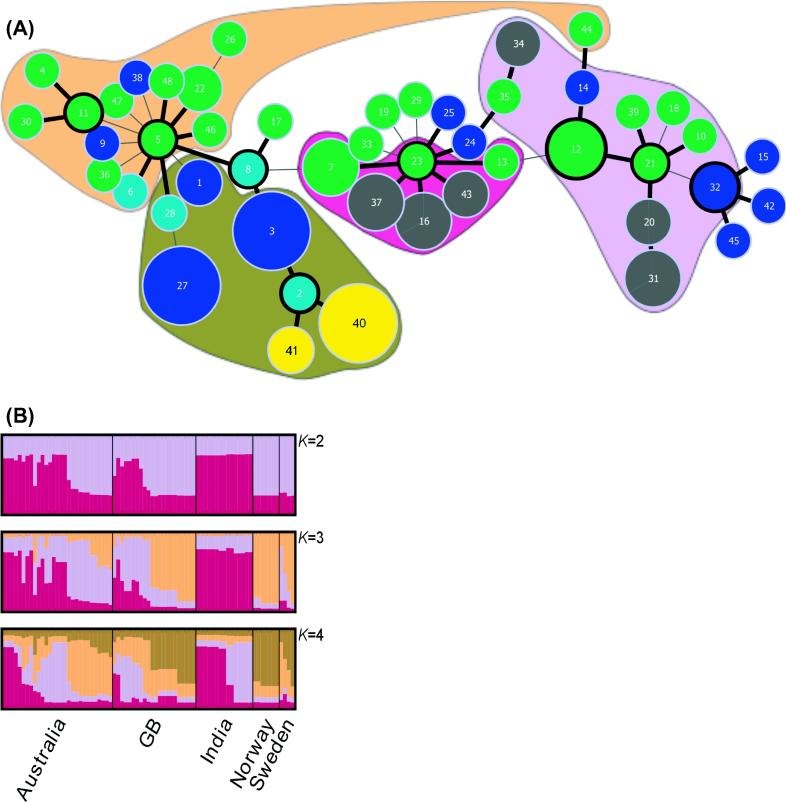
Diversity of global *D. nodosus* populations. (A) Minimum-spanning tree of *D. nodosus* MLVA data. Numbers indicate MLVA types and circle size is proportional to the numbers of isolates of each type (range 1–5). Single locus variants are connected by thick black lines and double locus variants by narrow grey lines. Circle colours indicate the country each MLVA type was isolated from: Australia (green), GB (dark blue), India (grey), Norway (yellow), Sweden (light blue); putative ancestral MLVA types have a thick black border. MLVA type membership of Structure clusters is highlighted in maroon (Cluster I), light purple (Cluster II), orange (Cluster III) and brown (Cluster IV). (B) Distruct plots of Structure output from *K *= 2 (upper image) to *K *= 4 (lower image) following analysis of 77 isolates of *D. nodosus*. Each isolate is represented by a single vertical line indicating its membership in each of *K* independent clusters. Geographic populations indicated along the bottom are separated by vertical black lines.

Global optimal eBURST (goeBURST) analysis identified five clonal complexes (CCs) and 11 singletons at the SLV level (Fig. 1A); the inclusion of double locus variants led to the formation of a large single group (Fig. 1A). At the SLV level, one CC contains isolates from Australia (CC11) and another only isolates from GB (CC32); only *pgrB* is present in the isolates within these CCs. The remaining three complexes (CC2/5/8, CC12/21 and CC23) contain isolates from Australia and GB, *pgrA* and *pgrB* variants are present in all three CCs. Isolates from India are present in two CCs (CC12/21 and CC23) and isolates from Scandinavia are only present in CC2/5/8. The Indian isolates originated from four districts of Jammu and Kashmir State, spread along a North–South cline from Bandipora in the north, through Ganderbal and Srinagar, to Anantnag in the south. There is some geographic differentiation between isolates, those from Bandipora and Ganderbal are a single MLVA type [31] only present in CC12, whereas of the 12 isolates from Anantnag and Srinagar, ten are in CC23 and two [MLVA type 20] are in CC12.

The clustering of individual MLVA types within CCs, and between CC connections is broadly supported by investigation of population structure using Structure (Fig. 1A and 1B). At the most hierarchical level, Structure analysis indicates there are seven independent populations of *D. nodosus* (Fig. S1). However examination of the results suggests that four populations is a more realistic interpretation of our data. The standard deviation (SD) of the mean estimate of the log probability of *K* is increased where *K* > 7, indicating a lack of confidence in cluster assignment at these values of *K*, and causing the peak in *ΔK* at *K* = 7. This suggests that there is greater confidence in cluster assignment where *K* < 7, and that the *ΔK* peak at *K* = 4 is the true number of detectable clusters in our data. Support for *K* = 4 is found by examination of the mean log probability (maximal at *K* = 4; Fig. S1); and by analysis of *Q* values at *K* = 4–6. Using a *Q* value threshold of 0.5, at *K* = 4, 67 of 77 isolates can be assigned to clusters (arbitrarily named I, II, III, and IV), which is in broad agreement with the goeBURST results (Fig. 1A and 1B). Cluster I contains the majority of CC23; two MLVA types distantly related to the inferred ancestral strain are assigned to cluster II. This second cluster contains isolates from CC12/21 and CC32. Cluster III contains isolates from CC5 and CC11, and one MLVA type assigned to CC12; and cluster IV predominantly consists of isolates from CC2 (Fig. 1). At *K* = 5 where *Q* > 0.5, 22 singletons are identified and only one MLVA type [27] is assigned to the putative fifth cluster; at *K* = 6, 34 singletons are identified and no MLVA types are assigned to the sixth cluster.

The $I_{A}^{S}$ value for the 77 strains was 0.0927, which differed significantly from zero (*P* < 10^−3^) indicating that recombination has played a key role in allelic distribution within *D. nodosus*. At the country level, $I_{A}^{S}$ values of 0.2160, 0.2753 and 0.3315 were detected in Australian, Indian and British populations respectively (all *P* < 10^−3^). The formation of a network structure (Fig. S2) following phylogenetic network analysis of pairwise distances between allelic profiles provides further evidence that recombination has influenced the evolution of *D. nodosus*. In contrast, CC11 and CC32, detected in one geographical location each (a single farm in GB and Australia respectively), both form tree-like structures in the network analysis, indicating clonal expansion.

## 4 Discussion

The aim of this study was to identify polymorphic loci in the *D. nodosus* genome and to use these to characterise a collection of global isolates. We identified globally widespread CCs, indicative of a core of *D. nodosus* MLVA types that are present in a variety of environments. Other CCs present only in certain countries have possibly evolved locally. This theory of core MLVA types, augmented by localised variants, is supported by the phylogenetic analyses where CC2, CC11 and CC32 contain isolates from individual regions that cluster at the ends of branches rather than appearing as major nodes; as is the case for CC5/8, CC23 and CC12/21, which contain isolates from several geographical locations (Fig. 1A).

The similarity in MLVA types detected in Australia and GB highlights a close relationship between isolates from these countries. It is likely that footrot was present in GB before it was present in Australia, given that sheep and footrot were present in the UK for many years before Australia was colonised (Youatt, 1837) and that sheep imported from the UK took *D. nodosus* with them. However, Australia is likely to have received multiple introductions of *D. nodosus* from separate sources e.g. Australia imported Merino sheep from Spain. In addition, many states in Australia have had control and elimination programmes for many years (Egerton et al., 2004; Mitchell, 2003) that are likely to have influenced the contemporary population of *D. nodosus*. This may explain why not all isolates from Australia are present in GB, and vice versa (as well as the fact that our sample of isolates is unlikely to be a complete representation) and why Pgr type and protease test results differ between the two countries (Table 1). Notwithstanding this difference in Pgr and protease test results between Australia and GB, the reported correlation between Pgr status and protease thermostability (Calvo-Bado et al., 2011) is confirmed here.

MLVA types from India, Norway and Sweden were present in the three main multi-country CCs. However, isolates from India and Scandinavia were never present in the same CC, nor Structure cluster, suggesting that isolates from these countries have different origins. According to both Structure and goeBURST, the Indian isolates are most similar to those from Australia. Footrot has only recently been detected in India, and its source can be attributed to the introduction of sheep from Australia, as part of a genetic improvement programme to upgrade wool quality in native Indian sheep breeds (Acharya, 1982). The geographical distribution of the two distinct populations of *D. nodosus* in India might be due to independent introductions of *D. nodosus*, or a single introduction of multiple types. The putative hybrid population [MLVA type 34], only present in isolates from Srinagar, might be a result of transhumance as flocks are moved between summer and winter pastures; during this time many flocks mix together and this might lead to the development of novel MLVA types. The detection of a DNTR19 allele unique to India (allele02) within this putative hybrid population suggests localised evolution may be taking place. Notwithstanding the potential for sampling bias, the lack of diversity at the DNTR09 locus in Indian isolates may reflect the strains introduced to the country, or indicate a lack of fitness of other alleles in Indian conditions. Continued surveillance of Indian and more widespread Asian isolates might reveal further insights into *D. nodosus* evolution and diversity.

The isolates from Scandinavia were assigned to CCs and clusters that also contained isolates from GB; possibly indicating that GB was the source of *D. nodosus* in Scandinavia. Equally there might be uncharacterised intermediates or a common, as yet unidentified, source for all of Europe. The first case of footrot in Sweden was detected in 2004 (Olofsson et al., 2005). In Norway footrot was eliminated in 1949 (Øverås, 1994) however the disease was detected again in 2008 (Meling and Ulvund, 2009). The apparent timing of these outbreaks, the restricted diversity at DNTR09 in Norwegian isolates compared to Swedish isolates and the presence of Norwegian isolates at terminal nodes and Swedish isolates at internal nodes on the minimum spanning tree indicates that footrot was probably present in Sweden before Norway. It is possible that the Norwegian isolates might derive from Swedish populations, directly or indirectly, or that they share a common ancestor. However further analysis of additional isolates from a wider range of countries is required to test this hypothesis more fully.

On the basis of the current data and historical records, we can be confident in the geographical clustering of GB, Australian and Indian isolates. However the clustering of Scandinavian and GB isolates is more speculative, it is possible that the Scandinavian populations of *D. nodosus* are unrelated to GB isolates, and are more closely linked to populations from other Northern European countries. It is also possible that footrot in Scandinavian sheep may have developed following recrudescence of existing bacterial populations because of changes in local environmental conditions. More isolates from more countries might clarify this.

Our results indicate that *D. nodosus* has evolved through a balance of recombination and clonal expansion events. Globally *D. nodosus* is characterised by a weak clonal population structure consisting of geographic sub-populations subject to genetic drift and within population recombination, but there is limited recombination between geographically isolated populations. The broad agreement in total number and membership of clusters/CCs between Structure (four clusters) and goeBURST (five CCs) gives our results repeatability and robustness, in addition, the detection of clonal groups agrees with earlier population analyses of *D. nodosus* (Buller et al., 2010; Ghimire and Egerton, 1999; Zakaria et al., 1998; Zhou and Hickford, 2000). However both our analyses identified a number of non-matching singletons suggesting, unsurprisingly, that there is greater global *D. nodosus* diversity than characterised in this report. An alternative interpretation may be that the detected singletons are short-lived intermediates or mutations that were isolated by chance.

The MLVA scheme described here is simple to use, transferable between laboratories, and has all the attributes required of a typing method suitable for use as an epidemiological tool to study the global distribution and diversity of *D. nodosus*. It has high discriminatory power (*D* > 0.95), typeability, reproducibility and epidemiological concordance (van Belkum et al., 2007). The level of MLVA discrimination is comparable to that achieved by PFGE of 796 Australian isolates [Simpson’s *D *= 0.98] (Buller et al., 2010), and greater than PFGE of 12 Malaysian isolates [*D *= 0.86] and PCR-RFLP of 66 isolates [*D *= 0.87; values calculated based on data presented] (Ghimire and Egerton, 1999; Zakaria et al., 1998). The variation in diversity at the four chosen loci is similar to that detected in a MLVA scheme for *S. agalactiae* [range of Simpson’s *D *= 0.47–0.90] (Haguenoer et al., 2011) which is used in epidemiological surveillance, supporting the proposal that the MLVA scheme developed here is suitable for epidemiological investigations. One potential drawback of the scheme is the repeat motif length of DNTR02 (5 bp). This is too small to accurately size on an agarose gel, so amplicons must either be sequenced, or amplified with a fluorescent-labelled primer and sized by fragment analysis, adding to analysis costs.

Loci with few alleles probably represent genomic regions that are slow to evolve, whereas those with greater diversity are probably evolving more rapidly. The locus with the greatest diversity in the scheme (DNTR02) has the smallest tandem repeat unit (5 bp) and the locus with the least diversity has the largest (108 bp). The high diversity at DNTR02 might be the result of an increase in slipped strand mispairing in small (1 – 10 bp) repeat units (Levinson and Gutman, 1987). In contrast, the seemingly restricted diversity at DNTR09 and DNTR19 might be due to location in a genomic region subject to selective pressures that lead to reduced extant variation. The lack of any relationship between MLVA type and serogroup indicates that the four selected loci are not located close to the *fimA* gene.

## 5 Conclusions

In conclusion, we have developed an MLVA typing scheme for *D. nodosus* with high discriminatory power, and have demonstrated its usefulness in a geographical epidemiological investigation. We identified clonal complexes containing isolates from one, two or three continents. The pattern of isolates within CCs suggests that GB and Australia share common *D. nodosus* isolates, that those from India originated from Australia and that those from Scandinavia originated from GB. Further, the isolates from India and Scandinavia are distinct. Additional studies are required to determine the usefulness of the *D. nodosus* MLVA scheme for surveillance.

## Figures and Tables

**Table 1:** Summary of *D. nodosus* isolate characteristics by country.

Country	Isolates (*n*)	Protease thermostability		Pgr		Serogroup								
		Thermostable	Thermolabile	A	B	A	B	C	D	E	F	G	H	I
Australia	29	10	19	10	19	7	6	3	1	1	5	2	1	3
GB	22	18	4	12	10	10	1	7	0	1	0	0	3	0
India	15	15	0	15	0	0	9	0	0	5	0	1	0	0
Norway	7	7	0	7	0	3	0	0	0	0	0	0	0	4
Sweden	4	0	4	1	3	1	0	1	0	1	0	1	0	0

**Table 2:** Diversity index (Simpson’s *D*) of individual and combined MLVA loci.

Locus	Number of alleles/types	Simpson’s diversity [*D*] (95% CI)
DNTR02	23	0.937(0.926–0.949)
DNTR09	4	0.504(0.434–0.573)
DNTR10	10	0.801(0.766–0.836)
DNTR19	4	0.691(0.640–0.741)
All loci	48	0.969(0.961–0.978)

**Table 3:** Allelic distribution of DNTR09 and DNTR19 by country.

Origin	DNTR09 alleles (*n*)				DNTR19 alleles (n)			
	02	04	05	06	02	03	04	05
Australia	2	11	15	1	0	8	9	12
GB	0	14	8	0	0	5	7	10
India	0	0	15	0	7	8	0	0
Norway	0	0	7	0	0	0	0	7
Sweden	0	2	2	0	0	0	0	4

## Appendix A Supplementary data

Supplementary data 1 This document file contains Supplementary materials.
